# Infraorbital Tuberculosis: A Case Report

**DOI:** 10.22038/ijorl.2020.43237.2422

**Published:** 2020-07

**Authors:** Rakesh Bambore-Suryanarayan-Rao, Bharathi Murundi-Basavarajaiah, Sreenivas-Kamath Kasaragod, Thanzeem Unisa

**Affiliations:** 1 *Department of ENT, JSS Academy of higher education and research (JSSAHER), Mysuru, Karnataka.*

**Keywords:** Extra pulmonary primary tuberculosis, Infraorbital tuberculosis, Sublabial endoscopic approach

## Abstract

**Introduction:**

Extra-pulmonary tuberculosis (EPTB) arising in extra-oral region in head and neck are rare, and when swellings arise from other sites such as infraorbital region, cheek, etc, tuberculosis is not usually considered for the differential diagnosis (DD) and often the diagnosis is missed and appropriate treatment is delayed.

**Case Report:**

We report a rare entity of primary tuberculosis, which presented as infraorbital swelling and our technique of performing sublabial approach to the swelling with endoscopic guided excision of the swelling and also we have review of literature of similar cases of primary tuberculosis presenting as swelling over the face over the past 5 year.

**Conclusion:**

Primary EPTB should be considered as DD in cases of chronic facial swelling.

## Introduction

Tuberculosis (TB) is a chronic granulomatous disease affecting human being, which is caused by mycobacterium tuberculosis, mycobacterium bovis and other atypical mycobacterias. Primary TB affects the lungs, lymph nodes, meninges, joints, skin and other tissue of the body. When it comes to extra pulmonary tuberculosis, we often face challenge in its diagnosis as well as for its management. The incidence of primary tuberculosis of oral cavity is reported as 0.05-5%.(1) Among the oral cavity tuberculosis, tongue is the most commonly reported site of lesion within the oral cavity(2).

Extra oral cases of primary tuberculosis is rare and often present as fistulas, ulcers, skin lesions. To our best knowledge, we present a unique site of primary tuberculosis and a method of its management.

## Case Report

52 year old female presented to the department of otorhinolaryngology with complain of unilateral gradually progressive swelling over the left side of the nose below the eyes since 2 months. The swelling was painless to begin with, but patient gives history of pain over the swelling since 2 weeks. 

There are no aggravating or relieving factors. No history of radiation of pain. No history of recent trauma. Patient also gives history of watering of left eye since 2 days. Not associated with nasal obstruction, nasal discharge. No history of visual disturbance. There was no history of significant weight loss or loss of appetite. No significant medical history (past history of tuberculosis, exposure to patients with tuberculosis), or family history.

On examination she was conscious cooperative and well oriented to time place and person. Vitals were stable. 

A swelling measuring 1.5 x 2 cm was noticed on the lateral aspect of the nasal bridge, inferior to the infraorbital ridge. Firm in consistency, irregular surface, ill-defined margins, non-tender, mobile in horizontal and vertical direction, no local rise of temperature, no skin changes and skin was pinch able. 

Rest of the ear, nose, throat and neck examination were within normal limits. No palpable neck lymph nodes.

On investigation

Blood parameters were within normal limits except for ESR, which was 76mm/hr.

HIV, HbsAg and HCV were negative.

**Fig1:**
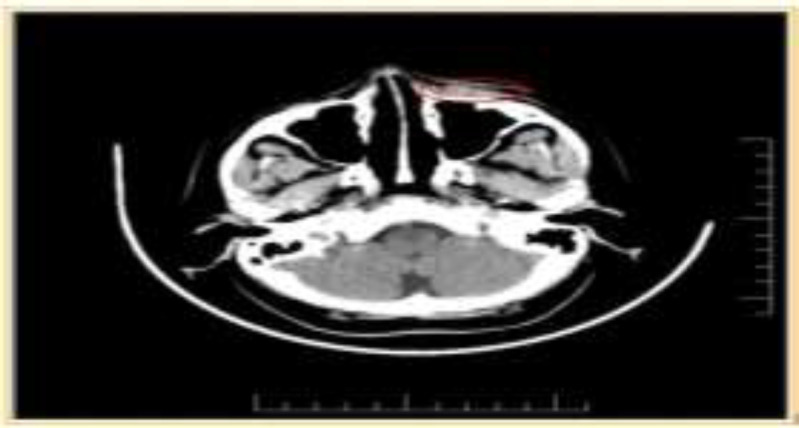
Showing CT axial cuts of lesion site, marked with red circle is the lesion present on left infraorbital region

FNAC was done from the lesion which showed multiple inflammatory cells- suggestive of chronic inflammation. A plain CT scan was done which showed- soft tissue density noted adjacent to anterior wall of left maxillary sinus. The lesion is not involving the anterior wall of sinus. (Fig.1).

*Chest x-ray PA view was normal*

**Fig 2:**
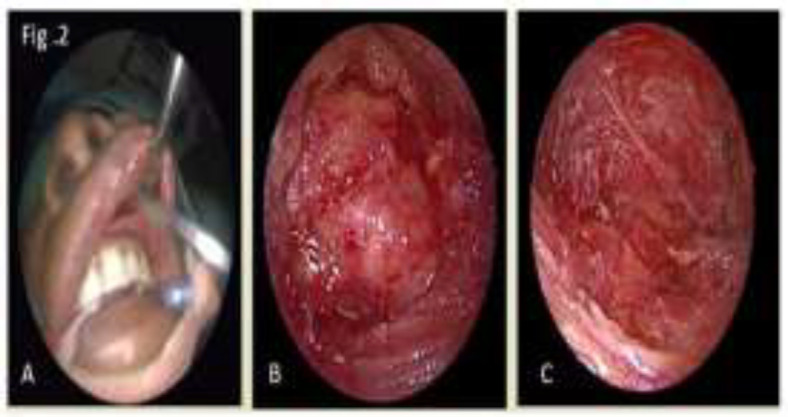
From left to right A- image showing the sublabial approach, B- Endoscopic visualisation of the lesion, C- Surgical bed of lesion after its total excision

Patient was planned for excision of the lesion under ga and was taken up for surgery after anaesthetic pre-op fitness. A cosmetically better approach was planned for this case i.e endoscopic sublabial approach to the swelling. Lesion was identified, it was a well encapsulated lesion present over the inferior orbital margin extending 1cm from the medial canthi laterally to the root of the nose. Lesion was removed in toto and sent for histopathology (Fig.2).

**Fig 3:**
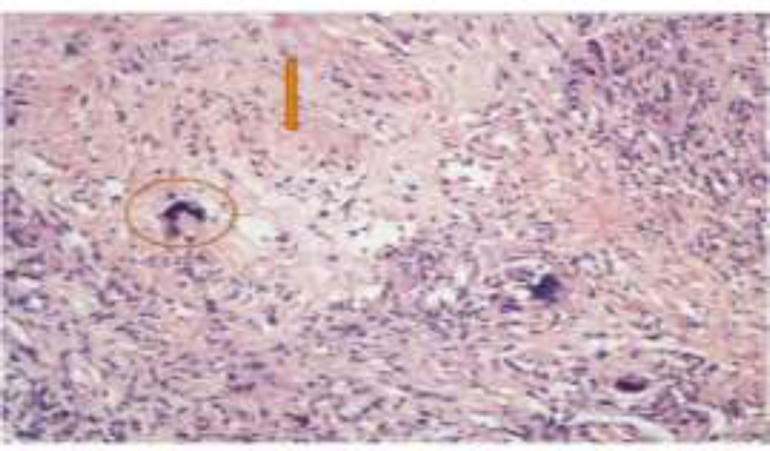
Showing the haematoxylin and eosin stained histopathology slide. Marked circle showing the epithelioid cells and arrow showing the caseation

 The histopathology showed epithelioid cells with caseation suggestive of tubercular pathology (Fig .3).

Later the patient was subjected to Mantoux test and sputum for acid fast bacilli and was found negative for both test. Patient was started on category 1 antitubercular treatment. And patient has successfully completed 4 months with good compliance and no recurrence of swelling post-surgery.

## Discussion

Extra pulmonary tuberculosis (EPTB) is described when the causative organism- Mycobacterium tuberculosis affects organs other than lung. It can affect any organ isolated or along with the primary foci in lung. The spectrum of EPTB can range from fatal illness when it affects the pericardium or meninges to benign lesions such as scrofula or lymphadenitis. The burden of the EPTB is 15-20% of the total tuberculosis cases. While in HIV cases it accounts for almost 40-50% of the cases. Among the estimated 2.1 million cases of TB around 16 percent that is equating to 3,36,000 cases were EPTB (3). When TB is screened, it is more often aimed at pulmonary TB. However we should also understand that EPTB also has a significant impact over the suffering patient thus is important to diagnose and treat the patient with antitubercular medications. EPTB when encountered is a diagnostic as well as a therapeutic challenge.As per the global TB report 2017 the estimated burden of TB in India was approximately 28 00,000 accounting for about quarter of the world TB cases. And the incidence of patient with HIV with TB is 87,000 i.e about 3% of total TB cases (4).With the implementation and widespread coverage of the universal immunisation programme, which has made BCG vaccination mandatory coupled with the effective public health care and public awareness, has aided in lowering the incidence of pulmonary TB over the past few years. However due to concurrent pandemic of human immunodeficiency virus infection and acquired immunodeficiency disease syndromes (AIDS) has brought about rise in the EPTB incidence, probably due to the increased risk of haematogenous dissemination of mycobacteria. EPTB burden is approximately 10% of all the TB cases ( this percentage can vary from region to region).the EPTB is found to occurs in lymphatics (40%), pleural (20%) bones and joints(10%) and rest of the rare sites account for (30%). Cutaneous TB accounts for about 1% of EPTB(5).The tuberculosis of cheek is very rare and often is secondary to the primary elsewhere in the body. The pathogenesis of this variant of tuberculosis remains so far unclear. A search was made on Pubmed search tool using- “cheek tuberculosis, infraorbital tuberculosis.” A total number of 6 cases have been so reported with swelling of cheek as presentation of primary tuberculosis (Table.1,2). 

**Table 1:** Cases of Tuberculosis of cheek and its characteristics

**Sl** **No**	**Authors**	**Histopathological Findings**	**Mantoux test**	**Acid Fast Bacilli staining**	**Culture**	**Biopsy Technique**	**Imaging modality**	**Treatment modality**	**Recurrence**	**"Other foci of Primary TB**
1	Knezevic P et al (6)	Chronic granulomatous inflammation	Negative	Negative	Positive	Excision	Not done	ATT Cat 1	Nil	Nil
2	Namdev R et al(7)	Caseating granuloma	Negative	Positive	NA	Incision	USG	ATT Cat 1	Nil	Nil
3	Neena C et al(8)	Caseating granuloma	Positive	Negative	Positive	Excision	CT	ATT Cat 1	Nil	Nil
4	Saravanam PK et al(9)	Epithelioid granuloma with giant cells.	Negative	Positive	NA	Not done	CT	ATT Cat 1	Nil	Left side pleural effusion.
5	Karbach J et al(10)	Epithelioid granuloma with giant cells.	Negative	Positive	Positive	Excision	MRI	ATT Cat 1	Nil	Nil
6	Gupta M et al(11)	Epithelioid granuloma with giant cells.	Negative	Positive	NA	Excision	CT	ATT Cat 1	Nil	Nil

**Table 2:** Continuation of review of literature

**Sl.** **No**	**Authors**	**Age**	**Gender**	**Symptoms**	**Duration**	**Site of lesion**	**Size**	**Examination Finding**		**FNAC**
								Consistency	Tenderness	
1	Knezevic P et al (6)	46	Female	Swelling	NA	Cheek	NA	Firm	Absent	Chronic inflammation
2	Namdev R et al(7)	4	Female	Swelling	3 months	Cheek	7*3	Firm	Absent	Epithelioid cell granuloma
3	Neena C et al(8)	31	Male	Swelling	3 months	Cheek	4*4	Firm	Absent	Granulomatous lesion
4	Saravanam PK et al(9)	26	Male	Swelling	1 month	Cheek	4*2	Firm	Absent	Epithelioid macrophage with necrotic background
5	Karbach J et al(10)	23	Male	Fistula	2 months	Cheek	NA	Soft	Absent	Not done
6	Gupta M et al(11)	39	Female	Swelling	4 months	Cheek	2.5*2	Firm	Absent	Inconclusive

The diagnosis of EPTB can be difficult due to the absence of typical constitutional symptoms of TB. Microbiological tests are often inconclusive i.e FNAC and culture, because the cutaneous tuberculosis is paucibacillary (bcr). Which held true for our patient also FNAC was inconclusive and did not point towards TB. 

Histopathological finding are characteristic of TB but remains difficult to be differentiated from the other granulomatous diseases. In our case HPE report was suggestive of TB. 

MRI and CT often aid in differentiating TB from other benign lesions. In our case patient underwent CT which was suggestive of soft tissue density noted adjacent to anterior wall of left maxillary sinus. The lesion was not involving the anterior wall of maxilla. 

## Conclusion

 Even with active preventive measures, tuberculosis is still a burden to the society. There has been an increase in the incidence of EPTB among individuals with AIDS and other immunocompromised conditions. Of late few cases of primary tuberculosis of cheek have been reported. Thus we should routinely screen cases of cheek swelling for tuberculosis.
